# Supplementary material for: Empagliflozin-Enhanced Antioxidant Defense Attenuates Lipotoxicity and Protects Hepatocytes by Promoting FoxO3a- and Nrf2-Mediated Nuclear Translocation via the CAMKK2/AMPK Pathway
Source: Antioxidants (Basel). 2022 Apr 19;11(5):799. doi: 10.3390/antiox11050799 (PMC9137911; doi:10.3390/antiox11050799)
Supplement: Supplementary file 1 [file antioxidants-11-00799-s001.zip › antioxidants-1613095-supplementary.pdf]

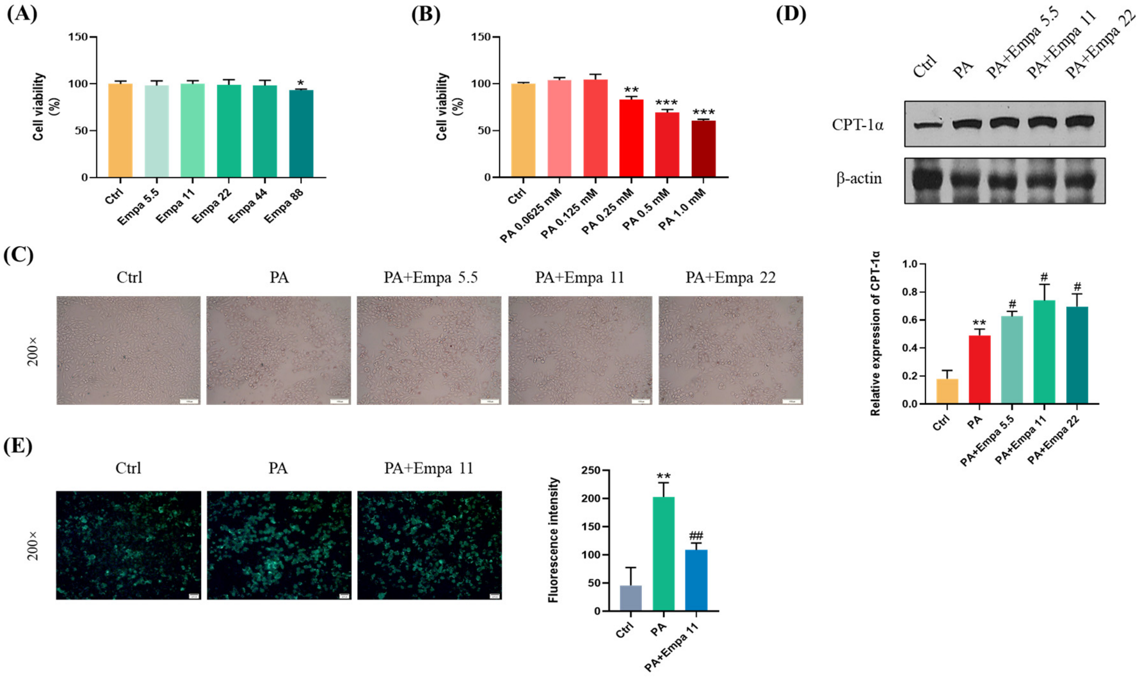

Figure S1. The cytotoxicity of Empa and PA in LO2 cells and the effects of Empa on lipid metabolism and intracellular Ca^{2+} concentration in the PA-treated LO2 cells. (**A**) Effect of Empa (5.5-88 μM) on cell viability of LO2 cells, and the result was analyzed by MTT assay. (**B**) PA showed significant cytotoxicity at concentrations above 0.25mM in LO2 cells. (**C**) Intracellular lipid content was assessed by Oil red O staining, and the result showed that Empa had no significant effect on lipid accumulation in PA (0.5mM)-treated LO2 cell lines. (**D**) Empa treatment further significantly increased CPT-1α expression in LO2 cells. (**E**) Empa remarkably dampened intracellular Ca^{2+} concentration in PA-treated LO2 cells. All data are expressed as mean ± SD (n=3). *$p < 0.05$, **$p < 0.01$ and ***$p < 0.001$ versus Ctrl. #$p < 0.05$ and ##$p < 0.01$ versus PA. Ctrl, PA-untreated normal control group; Empa 5.5-88, Empa-treated (at final concentrations of 5.5-88 μM) control group; PA, PA-treated control group; PA+Empa 5.5-22, PA-treated and Empa-treated (at final concentrations of 5.5-22 μM) groups.

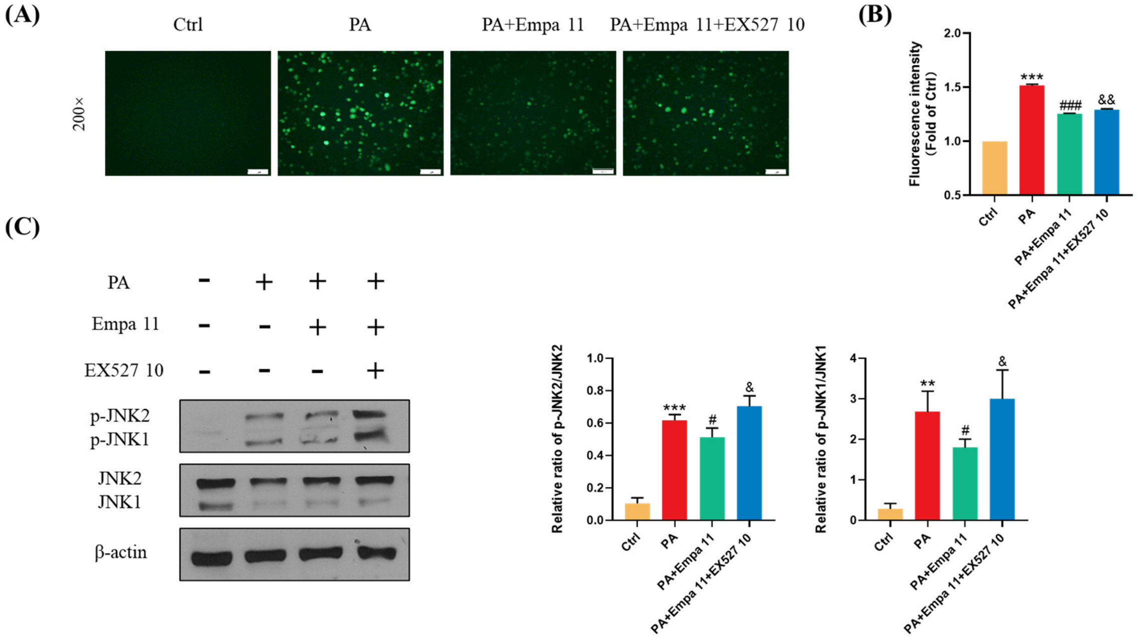

Figure S2. EX527 increased cellular ROS level and the phosphorylation of JNK in LO2 cells. (**A**,**B**) EX527 (10 μM) increased intracellular ROS production in PA-treated and Empa-treated LO2 cells. (**C**) EX527 significantly upregulated phosphorylation level of JNK1 and JNK2 in PA-treated and Empa-treated LO2 cells. All data are expressed as mean ± SD (n=3). **p < 0.01 and ***p < 0.001 versus Ctrl; #p < 0.05 and ###p < 0.001 versus PA; &p < 0.05 and &&p < 0.01versus PA+Empa 11. Ctrl, PA-untreated normal control group; PA, PA-treated control group; PA+Empa 11, PA-treated and Empa-treated (at final concentrations of 11 μM) group; PA+Empa 11+EX527 10, PA-treated, Empa-treated (at final concentrations of 11 μM) and EX527-treated (at final concentrations of 10 μM) group.

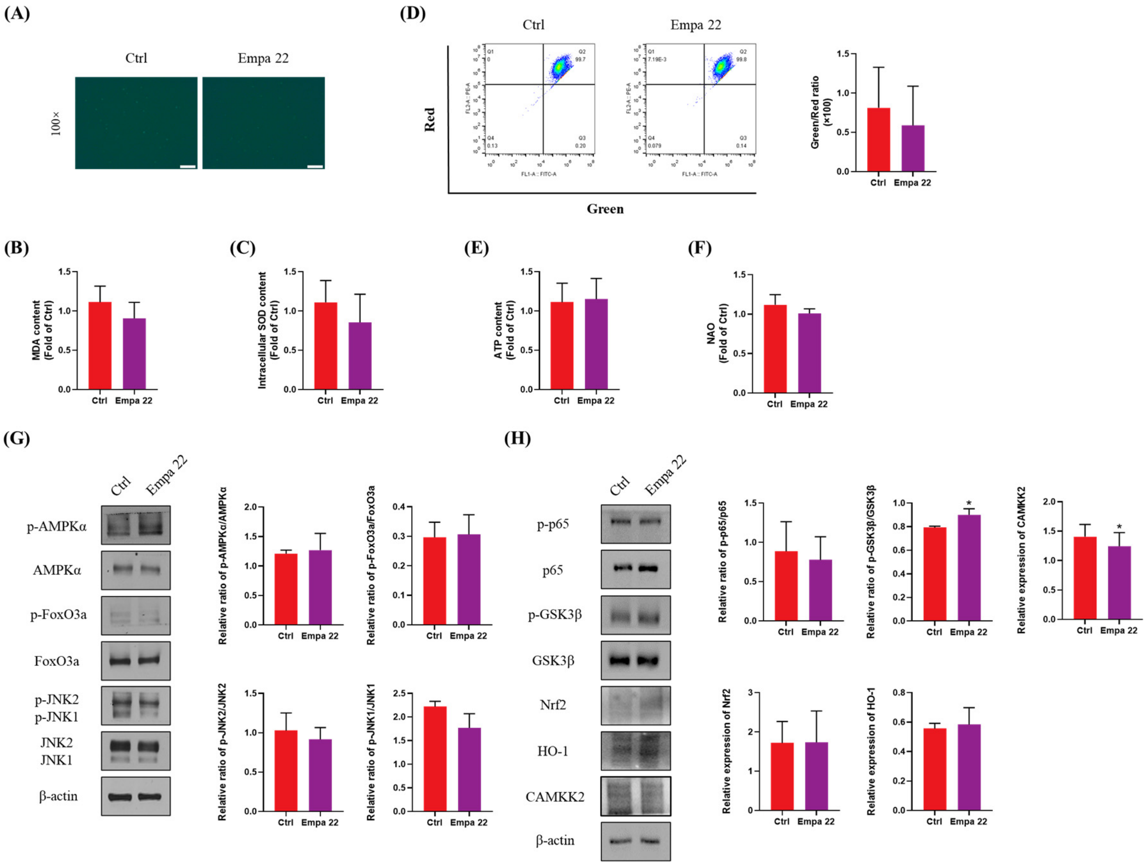

Figure S3. Effects of Empa on normal LO2 cell lines. (**A**) Effect of Empa (22 μM) on ROS generation in PA-untreated LO2 cells. (**B**) Effect of Empa (22 μM) on MDA content in PA-untreated LO2 cells. (**C**) Effect of Empa (22 μM) on SOD content in PA-untreated LO2 cells. (**D**) Effect of Empa (22 μM) on mitochondrial membrane potential in PA-untreated LO2 cells. (**E**) Effect of Empa (22 μM) on ATP production in PA-untreated LO2 cells. (**F**) Effect of Empa (22 μM) on mitochondrial mass in PA-untreated LO2 cells. (**G**) Effects of Empa (22 μM) on the phosphorylation of AMPKα, FoxO3a, JNK1 and JNK2 in PA-untreated LO2 cells. (**H**) Effects of Empa (22 μM) on the phosphorylation of p65 and GSK3β as well as the protein expression level of Nrf2, HO-1 and CAMKK2 in PA-untreated LO2 cells. All data are expressed as mean ± SD (n=3). *p < 0.05 versus Ctrl. Ctrl, normal control group; Empa 22, Empa-treated (at final concentrations of 22 μM) control group.
